# Oral administration of propionic acid during lactation enhances the colonic barrier function

**DOI:** 10.1186/s12944-017-0452-3

**Published:** 2017-03-23

**Authors:** Zhaobo Xia, Yijiang Han, Ke Wang, Shikun Guo, Dazhou Wu, Xiaozhong Huang, Zhongrong Li, Libin Zhu

**Affiliations:** 1 Department of Pediatric Surgery, the Second Affiliated Hospital & Yuying Children’s Hospital of Wenzhou Medical University, Xueyuan West Road, 109#, Wenzhou, Zhejiang 325000 China; 2 Department of Pediatric Surgery, the First Affiliated Hospital of Wenzhou Medical University, Baixiang South, Wenzhou, Zhejiang 325000 China

**Keywords:** Propionic acid, Barrier function, Tight junction, Ussing chamber, Gas chromatography

## Abstract

**Background:**

Propionic acid is a three-carbon short chain fatty acid (SCFA) that has various effects on colonic functions. Although several studies have shown the effects of propionic acid on intestinal mucosal barrier function, studies of the promotion effect during pre-weaning are rare in the literature as far as we know.

**Methods:**

Pre-weaning male Sprague-Dawley rats 7 days after birth were given an oral 0.2 mL/10 g of 200 mM propionic acid solution in the propionic acid group or normal saline solution in the control group by gavage twice a day for ten days. The proximal colonic contents were used for extraction and determination of propionic acid by gas chromatographic analysis; the transepithelial electrical resistance (TER) of colonic tissue was detected by an Ussing chamber; the alterations of ZO-1, Claudin-1, Claudin-8 and Occludin proteins were analyzed by Western blot and immunohistochemistry; and The activity of ERK and p38 MAPK was determined by the phosphorylation status of ERK1/2 and p38 with Western blot.

**Results:**

Our results suggested a higher concentration (23.5 ± 1.9 mmol/kg) of propionic acid compared to the physiological concentration (18.1 ± 0.9 mmol/kg) in colonic contents after oral administration increased the value of TER and the expression of ZO-1, Claudin-1, Claudin-8 and Occludin compared to the control group. Furthermore, the expression levels of phosphorylated ERK1/2 and p38 MAPK were increased in propionic acid group.

**Conclusions:**

We concluded that continuous oral administration of propionic acid during lactation may increase its concentration in the proximal colon and promote epithelial barrier function of proximal colon by enhancing the expression of ZO-1, Claudin-8, Claudin-1 and Occludin via increases in the expression of ERK1/2 and p38 MAPK.

## Background

Short chain fatty acids (SCFAs), primarily acetic acid, propionic acid and butyric acid, are mainly produced by the fermentation of fibers and resistant starches that require the presence of specific colonic bacteria [1]. There is now an abundance of evidence to indicate that SCFAs play an important role in the maintenance of health and the development of disease [2, 3]. It has been confirmed that SCFAs can not only serve as the principal energy source for colonic epithelia, but they can also reduce the incidence of colonic inflammatory disease by inhibiting inflammatory cytokine release [4, 5]. In particular, SCFAs are considered to play an important role in anti-tumorigenic activity by inhibiting cancer cell proliferation and inducing apoptosis [6, 7].

The barrier function of colonic mucosa plays a key role in maintaining the balance between host and intestinal microbes. Tight junctions (TJs) as the critical physical intestinal barrier are the principal determinant of mucosal permeability [8]. Occludin, claudins and junctional adhesion molecules are the major transmembrane proteins linked to the cytoskeleton by zonula occludens (ZO), and they constitute the TJ [9]. The intestinal TJ seals the space between adjacent epithelia to effectively prevent pathogenic bacteria, endotoxins and other harmful substances from crossing the mucosa to the blood and the abdominal cavity [10].

SCFAs can dose-dependently suppress the permeability of isolated colonic mucosa and the Caco-2 cell monolayer in the short term in vitro [11, 12]. However, most of the literature focuses on butyrate and to a lesser extent on acetate. Consequently, the potential effects of propionic acid have long been underestimated [13]. In addition, pre-weaning is a special period in which the intestinal mucosal barrier has not yet matured, which results in the lack of resistance to various stresses [14]. Therefore, the pre-weaning rats were given an oral 2 mL/10 g of 200 mM propionic acid solution by gavage, and our study demonstrated that the oral administration of propionic acid during lactation increased its concentration in the proximal colon and enhanced epithelial barrier function.

## Methods

### Materials and antibodies

Phosphate-buffered saline (PBS) was purchased from Solarbio (Life Sciences Co. Ltd, Beijing, China). acetic acid, propionic acid and butyric acid, ethyl acetate, D-Mannitol, D-(+)-Glucose and agarose were purchased from Sigma (St Louis, MO, USA). Polyclonal rabbit antibodies for ZO-1, Claudin-8, Claudin-1 (Invitrogen Inc., USA), Occludin (Proteintech, Hubei, China), p38 MAPK, phospho-p38 MAPK, ERK1/2, phospho-ERK1/2 (Cell Signaling Technology, USA) and the monoclonal mouse antibody for glyceraldehyde 3-phosphate dehydrogenase (GAPDH) (Bioworld Inc., USA) were used as the primary antibodies. Horseradish peroxidase (HRP)-conjugated goat anti-rabbit IgG antibodies and HRP-conjugated goat anti-mouse IgG antibodies (Bioworld Inc., USA) were used as secondary antibodies. The DAB Detection Kit (Polymer) (ZSGB-BIO, Beijing, China) was used for immunohistochemistry. SuperSignal West Dura Extended Duration Substrate (ThermoFisher, USA) was applied for chemiluminescence.

### Animals

All of the experimental procedures were performed in compliance with the ethical principles of animal welfare. Pre-weaning male Sprague-Dawley rats (Laboratory Animal Centre, Wenzhou Medical University, China) 7 days after birth (18–20 g) were randomized into the control and propionic acid groups. The rats were given an oral 0.2 mL/10 g of 200 mM propionic acid solution in the propionic acid group or a normal saline solution in the control group by gavage twice a day for ten days. On the 11th experimental day, all of the rats were anesthetized and sacrificed to collect tissue and the contents of the colon.

### Gas chromatographic analysis

Each gram of colon contents was thawed and suspended in 5 mL of water and homogenized with a vortex for 2 min, followed by centrifugation (>10,000 r/min) for 10 min. Next, 0.1 mL of 0.5 M sulfuric acid was added to the supernatant of the colon content solution per milliliter to adjust the pH. Each milliliter of supernatant was extracted with 1 mL of ethyl acetate by vortex mixing for 2 min, centrifuged (>10,000 r/min) for 10 min and then incubated at 4 °C for 30 min. The supernatant ethyl acetate extracts were stored at −20 °C before gas chromatography (GC) analysis.

Chromatographic analysis was performed using an Agilent 6890 N GC system equipped with a flame ionization detector (FID) and an N10149 automatic liquid sampler [15], an HP-INNOWAX column, 30 m × 0.32 mm × 0.25 μm (Agilent, USA), was also used.

The temperature increase procedure was performed as follows: the initial temperature was 100 °C and was maintained for 0.5 min, then increased to 180 °C at 8 °C/min and maintained for 1 min, then increased to 200 °C at 20 °C/min, and finally maintained at 200 °C for 5 min. The temperatures of the injection port and FID detector were 200 °C and 240 °C, respectively. The flow rate of nitrogen was 20 mL/min with a split ratio of 1:10. 1 μL sample solution was injected for analysis. The SCFAs were identified by their specific retention times under the above GC conditions. Propionic acid calibration curve was made in the range of 0.75–6 mmol/L, and the concentration of the sample solution was converted to the propionic acid concentration in the colon contents.

### Transepithelial electrical resistance (TER) in the colonic wall using an Ussing chamber

Approximately 2 cm of colonic tissues were quickly taken from the rats within 5 min after anesthetization. Each specimen was opened along the mesenteric line and then rinsed with ice-cold 0.9% sodium chloride solution and kept in the same solution aerated with carbogen (95% O_2_–5% CO_2_) before being mounted in an Ussing chamber. Prepared colonic specimens were mounted on a 6-Channel EasyMount Ussing Chamber System (Physiologic Instruments, USA) that exposed a circular area of 0.031 cm^2^. Then, 5 mL of Manital-Krebs buffer was added to the side of the mucosa while 5 mL of Glucose-Krebs buffer was added to the serosa side. The Ussing chamber system was maintained at a constant temperature of 37 °C by using circulating water, and the medium on both sides was filled with carbogen (95% O2–5% CO_2_). Transepithelium electrical resistance (TER) was recorded for each specimen for 120 min after a 10 min stable period using the Ussing chamber technique.

### Western blot

After being excised and rinsed with ice-cold 0.9% sodium chloride solution, the proximal colons were homogenized by mechanical disruption in lysis buffer (50 mM Tris pH 7.4, 150 mM NaCl, 1% TritonX-100, 1% sodium deoxycholate, 0.1% SDS, 2 mM sodium pyrophosphate, 25 mM β-glycerophosphate, 1 mM EDTA, 1 mM Na_3_VO_4_, 0.5 mg/L leupeptin, 1 mM PMSF) and incubated on ice for 30 min. The lysates were centrifuged (10,000 r/min) for 30 min. The protein extracts were used to determine the quantity of total protein with the BCA method, and the extracts were boiled with SDS loading buffer and separated by 10% SDS–PAGE gels. After transferring the proteins to polyvinylidene fluoride membranes, the blotting procedure was performed as follows: the membranes were blocked in 5% nonfat milk in Tris Buffered Saline with Tween-20 detergent (TBST) for two hours at room temperature, incubated with primary antibodies (rabbit anti-Claudin-1, Claudin-8, Occludin, p38 MAPK, phospho-p38 MAPK, ERK1/2, phospho-ERK1/2, 1:1000 and mouse anti-GAPDH, 1:5000) overnight at 4 °C and then incubated with secondary antibodies for two hours at room temperature. The chemiluminescent signals of the bands were collected using the ChemiDoc MP system (Bio-Rad, USA). Quantification of protein intensity was performed using Image J software, and the results were expressed as the ratio of relative intensity of target proteins to the internal standard, GAPDH.

### Immunohistochemistry

Next, 4 μm sections were placed on clean, positively-charged microscope slides and heated in a tissue-drying oven for 2 h at 60 °C. The sections were washed in xylene 3 times for 5 min for deparaffinization and hydrated through a series of graded concentrations of alcohol. The slides were heated in 10 mM sodium citrate buffer at pH 6.0 at 100 °C for 20 min and cooled by incubation at room temperature in buffer for 20 min for antigen retrieval. Endogenous peroxidase activity was blocked with 3% H_2_O_2_ for 5 min. Subsequently, sections were blocked with BSA for 30 min and then incubated with the primary antibodies (rabbit anti-ZO-1, Claudin-8, 1:300) overnight at 4 °C. Afterwards, the sections were incubated with the biotinylated goat anti-rabbit IgG polymer for 20 min at room temperature. Immunoreactivity was visualized by diaminobenzidine, and the sections were counterstained with hematoxylin. Finally, the slides were dehydrated and mounted. A microscope (BX51, OLYMPUS, Japan) was used to examine the specimens.

### Statistical analysis

Statistical analyses were conducted using SPSS Statistics 23.0 (IBM Corporation, USA) and Prism 7.0 (GraphPad Software, Inc. USA) for Mac OS X. All of the data are shown as the means ± standard deviation (SD). Data on the TER were analyzed using a repeated measures analysis of variance (ANOVA), and the *t* test was used for the comparison between the two groups. *P* < 0.05 referred to significant differences.

## Results

### Administration of propionic acid by oral gavage enhanced the concentration in colonic contents

**Fig. 1:**
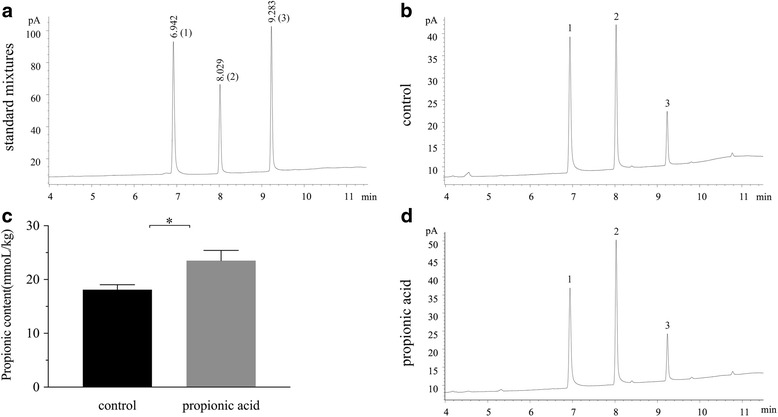
Gas chromatograms of standard mixtures (**a**) and proximal colon contents of the control group and propionic acid group (**b**, **d**). Peak identification: 1: acetic acid; 2: propionic acid; 3: butyric acid. Concentration of propionic acid (mmol/kg) in the proximal colon contents for ten days of oral gavage with a normal saline solution or 200 mM propionic acid in Sprague-Dawley rats (**c**). Each value was presented as the mean ± SD (*n* = 8), and significant differences between the groups are indicated as *P < 0.05 according to the results of a *t*-test

Figure 1a shows a typical chromatogram of a standard mixture containing the major SCFAs (acetic acid, propionic acid and butyric acid). The standard mixture was dissolved in ethyl acetate, and the peaks were identified by comparing retention times after injecting a series of graded concentrations of the individual SCFAs. To measure the concentration of propionic acid in the colonic contents, the calibration curve of propionic acid (y = 3.8431 × + 16.915, where (x) is the concentration (mM) and (y) is the peak area count) was performed and showed good linearity (R^2^ ≥ 0.997) for a wide range of concentrations (4–32 mM) that allowed the measurement of propionic acid in the proximal colon content samples. Figure 1b, d shows a chromatogram of SCFAs, particularly propionic acid in the colonic content samples of the control group and the propionic acid group. The difference of the concentration of propionic acid in the colonic contents between the oral administration of the normal saline solution and propionic acid by gavage is shown in Fig. 1c. The concentration of propionic acid in the control group (18.1 ± 0.9 mmol/kg) and the propionic acid group (23.5 ± 1.9 mmol/kg) was measured by the GC method, as previously described, and both concentrations were in the range of the physiological concentration in the proximal colon[16]. The propionic acid content in the proximal colon after the rats were given an oral 2 mL/10 g of 200 mM propionic acid solution by gavage twice a day was significantly higher than the control group (*P* < 0.05).

### Higher concentration of propionic acid enhanced intestinal epithelial barrier function

**Fig. 2:**
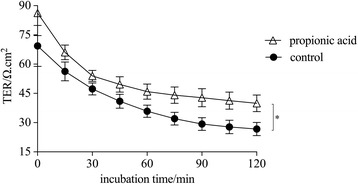
Transepithelial electrical resistance (TER) of proximal colonic specimens of propionic acid and the control group in vitro detected with Ussing chambers over a 120 min incubation time. Each value was presented as the mean ± SD (*n* = 9), and significant differences between groups are indicated as *P < 0.05 according to the results of a repeated measures analysis of variance (ANOVA)

TER was detected by the Ussing chamber system, which is a representation of intestinal epithelial barrier function [17], and a trend of slowing down for both the control and propionic acid groups over the 120 min incubation time in vitro was found. However, the TER was significantly higher after the rats received 200 mM propionic acid by oral gavage twice a day for ten days compared to treatment with normal saline solution at all time points, particularly the initial value at 0 min (propionic acid group: 86.2 ± 11.6 Ω.cm^2^, control group: 69.4 ± 10.5 Ω.cm^2^), as shown in Fig. 2 (*P* < 0.05).

### A higher concentration of propionic acid enhanced the intestinal barrier by increasing the expression of Claudin-1, Claudin-8 and Occludin

**Fig. 3:**
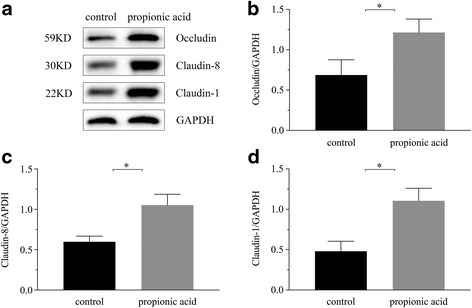
Western blots of Occludin, Claudin-8 and Claudin-1 in the proximal colon and glyceraldehyde-3-phosphate dehydrogenase (GAPDH) was performed as an internal standard (**a**). The relative density analysis of the control and propionic acid groups was summarized as the ratio of the relative intensity of target proteins to GAPDH (**b**, **c**, **d**). Each value was expressed as the mean ± SD (*n* = 10), and significant differences between the groups are indicated as *P < 0.05 according to the results of a *t*-test

Transmembrane elements, such as occludin and claudin, as well as the peripheral membrane protein ZO-1, are major components of the TJ. A ~22 kDa band for Claudin-1, ~30 kDa for Claudin-8 and ~59 kDa for Occludin appeared in the Western blot analyses and demonstrated changes in expression after the oral administration of 200 mM propionic acid compared to the control group. Figure 3a shows the signal bands of proteins detected at the expected molecular weights. The results of the proteins expression were summarized as a ratio of relative intensity of the target proteins to GAPDH, as shown in Fig. 3b, c, d. The expression of Claudin-1, Claudin-8 and Occludin in the proximal colon increased significantly compared to the control group (*P* < 0.05).

### ZO-1 and Occludin staining in proximal colon epithelia

**Fig. 4:**
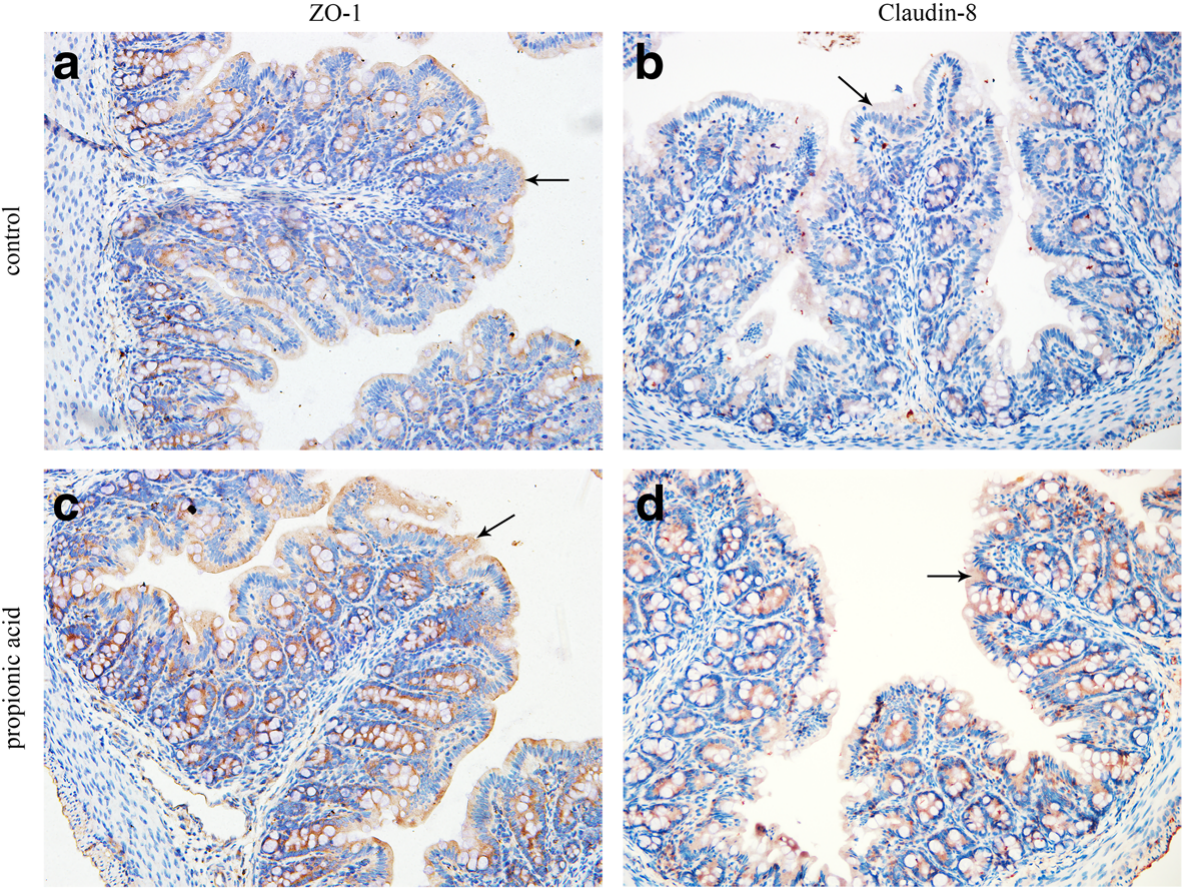
Two of the major tight junction proteins, ZO-1 and Claudin-8, were stained different grades of brown by immunohistochemistry (X 400). The brown staining of the proximal colonic epithelia lining villi in the propionic acid group (**c**, **d**) was greater compared to the control group (**a**, **b**)

Immunohistochemistry was performed to analyze the expression and distribution of TJs in the proximal colon by staining for the ZO-1 and Claudin-8 proteins. As Fig. 4 shows, ZO-1 and Claudin-8 were located at the superficial layer of the colonal mucosa connecting the epithelia and were consistent with TJ distribution. The staining intensity of ZO-1 and Claudin-8 in the propionic acid group (Fig. 4c, d) was significantly higher for both proteins compared to the control group (Fig. 4a, b) (*P* < 0.05), which agreed with the results of the Western blots. In addition, the enhancement of Claudin-8 expression was more obvious compared to that of ZO-1.

### Propionic acid enhanced the intestinal barrier via phosphorylation of ERK1/2 and p38 MAPK

**Fig. 5:**
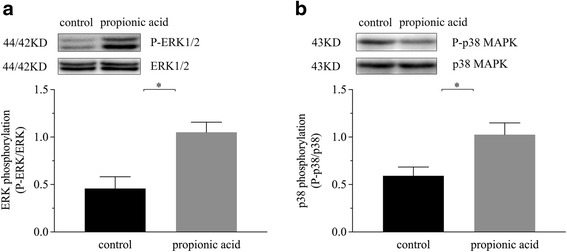
The expression of ERK1/2 and phospho-ERK1/2 (**a**), p38 MAPK and phospho-p38 MAPK (**b**) in the proximal colon and the phosphorylation status of ERK1/2 and p38 were summarized as the ratio of the relative intensity of P-ERK1/2 to ERK1/2 and P-p38 to p38, respectively. Each value was expressed as the mean ± SD (*n* = 10), and significant differences between the groups are indicated as *P < 0.05 according to the results of a *t*-test

We measured the changes in phosphorylation status of ERK1/2 (~44/42KD) and p38 (~43KD) after the oral administration of 200 mM propionic acid. Figure 5a, b shows the signal bands of proteins detected at the expected molecular weights and the expression levels of phosphorylated ERK1/2, p38 MAPK were significantly increased in the propionic acid group compared to the control group (*P* < 0.05).

## Discussion

During lactation, individuals are susceptible to intestinal diseases, such as necrotizing enterocolitis, because the intestinal mucosal barrier has not yet matured and there is a lack of resistance to various stressors [14]. Therefore, pre-weaning male Sprague-Dawley rats 7 days after birth, which simulated the neonatal period in humans, were used as models in our research. Their proximal colonic contents were extracted, and we determined the amount of propionic acid in the contents after the continuous oral administration of 200 mM propionic acid for ten days. The results of the GC analysis revealed that the propionic acid group had a greater propionic acid concentration in the proximal colon compared to the control group. Therefore, we discovered that the oral administration of 0.2 mL/10 g of 200 mM propionic acid by gavage increased the propionic acid concentration even though a majority of the propionic acid was absorbed by the small intestine [18].

In the physical environment, the primary amount of propionic acid was derived from the fermentation of undigested food by the colonic microbiota. Propionic acid has various functions in the colon as a major player in the maintenance of gut and immune homeostasis [19, 20]. The physiological concentration of propionic acid could immediately enhance barrier function of the colonic epithelium over a short period of time in vitro [21, 22]. As far as we know, studies of the long-term promotion effect of propionic acid in the colon in vivo, particularly for pre-weaning rats, are limited. To maintain the intact structure of the proximal colon epithelium [21], we detected the TER of whole colonic tissues with an Ussing chamber. The results showed that a higher concentration of propionic acid could promote proximal colonic barrier function in vivo, which was supported by greater TER values. The TJ is the most important structure in the mucosal barrier, and it plays a critical role in the maintenance of mucosal barrier function [23]. We assumed that a higher concentration of propionic acid may influence the expression of ZO-1, Claudin-8, Claudin-1 and Occludin, which are the major components of the TJ. The results of the Western blots demonstrated that the expression levels of Claudin-1, Claudin-8 and Occludin increased after the oral administration of 200 mM propionic acid for ten days; moreover, the immunohistochemistry of ZO-1 and Claudin-8 was consistent with the Western blot results.

Numerous studies have shown that under pathological conditions, such as inflammatory bowel disease and obesity, the increase in gut permeability is associated with much lower concentrations of SCFAs in the intestine [2, 24]. Oral supplementation of SCFAs after intestinal damage can suppress colonic epithelial permeability [25–27]. Furthermore, under physiologic status, the long-term promotive effect of a higher concentration of propionic acid on proximal colonic barrier function of pre-weaning rats was confirmed in our study.

Several molecular mechanisms by which SCFAs to modulate biological responses of the host have been proposed and elucidated. The major mechanisms involves the direct inhibition of histone deacetylases (HDACs) to directly regulate gene expression [28–30] and signaling through G-protein-coupled receptors (GPCRs) [31–33]. Propionic acid was the most potent and efficacious ligand for GPCR4 [34]. In this study, we showed that phosphorylated ERK1/2 and p38 MAPK were more strongly expressed in colonal mucosa after the oral administration of 200 mM propionic acid during lactation compared to the control group. As an intracellular mechanism, the ERK1/2 or p38 MAPK pathways is acknowledged as one of signals downstream of GPCRs [35–37]. It was previously reported that the ERK1/2 and p38 pathways are involved in the regulation of cell proliferation [38]. Therefore, our study suggested that ERK1/2 and p38 MAPK signaling pathways were involved in the propionic acid related promotion effect on colonic barrier function. But, the mechanism underlying the propionic acid activation of ERK1/2 and p38 MAPK remains to be further elucidated.

## Conclusions

We concluded that continuous oral administration of propionic acid during lactation may increase its concentration in the proximal colon and promote epithelial barrier function of proximal colon by enhancing the expression of ZO-1, Claudin-8, Claudin-1 and Occludin via increases in the expression of ERK1/2 and p38 MAPK. Our results also highlighted the potential value of propionic acid for the prevention and treatment of intestinal diseases, such as necrotizing enterocolitis, and the regulation of nutrition in newborns.

## Abbreviations

GAPDH: Glyceraldehyde 3-phosphate dehydrogenase
GC: Gas chromatography
GPCRs: G-protein-coupled receptors
HDACs: Histone deacetylases
SCFA: Short chain fatty acid
TER: Transepithelial electrical resistance
TJs: Tight junctions
ZO: Zonula occludens
